# Cross-Cultural Adaptation and Initial Psychometric Evaluation of the Adult Carer Quality of Life Questionnaire (AC-QoL) Among Informal Caregivers of Adults Receiving Home Mechanical Ventilation in Poland

**DOI:** 10.3390/jcm15103587

**Published:** 2026-05-07

**Authors:** Jakub Cichoń, Lucyna Płaszewska-Żywko, Zbigniew Szkulmowski, Maria Kózka

**Affiliations:** 1 Department of Specialized Nursing, Faculty of Health Sciences, Jagiellonian University Medical College, ul. Kopernika 25, 31-501 Kraków, Poland; 2 Department of Anaesthesiology and Intensive Care, Antoni Jurasz University Hospital No. 1, ul. Skłodowskiej-Curie 9, 85-094 Bydgoszcz, Poland; 3 Medowent, Center for Home Mechanical Ventilation, ul. Jagiellońska 103, 85-027 Bydgoszcz, Poland

**Keywords:** quality of life, informal caregivers, home mechanical ventilation, home care, long-term care, psychometric validation, cross-cultural adaptation

## Abstract

**Background/Objectives**: The increasing use of home mechanical ventilation in Poland highlights the need for reliable and culturally appropriate tools to assess caregiver quality of life. This study aimed to translate, culturally adapt, and evaluate the initial psychometric properties of the Polish version of the Adult Carer Quality of Life Questionnaire (AC-QoL) among informal caregivers of adults receiving home mechanical ventilation. **Methods**: A total of 203 informal caregivers participated. The cross-cultural adaptation followed standard procedures. Confirmatory Factor Analysis was conducted to assess the factor structure. Reliability and validity were assessed using Cronbach’s alpha, McDonald’s omega, Composite Reliability, Average Variance Extracted, and correlations with caregiver burden, perceived stress, and life satisfaction. **Results**: Confirmatory Factor Analysis supported the original eight-factor structure, demonstrating good model fit (CFI = 0.954; TLI = 0.950; RMSEA = 0.069; SRMR = 0.078), with factor loadings ranging from 0.64 to 0.96. The Polish version of the AC-QoL demonstrated high internal consistency (Cronbach’s α = 0.95). Convergent validity was confirmed by significant correlations with caregiver burden, perceived stress, and life satisfaction. **Conclusions**: The Polish version of the AC-QoL demonstrates promising psychometric properties and may support clinical assessment and research in home-based long-term care.

## 1. Introduction

The quality of life (QoL) is a multidimensional and complex concept. The World Health Organisation defines QoL as “*(…) individuals’ perception of their position in life in the context of the culture and value systems in which they live and in relation to their goals, expectations, standards and concerns*” [1] (p. 1403). Contemporary approaches emphasise the importance of subjective experiences across various aspects of life. QoL is therefore based on both quantifiable indicators and personal perceptions [2] and represents a balance between an individual’s internal state, social relationships, and environmental conditions [3].

Advances in respiratory support technologies and home care have led to a growing use of home mechanical ventilation (HMV) [4]. In Poland, the prevalence of HMV has increased over the years, reaching approximately 20 per 100,000 inhabitants [5]. The provision of home care is associated with several benefits for patients, including improvements in health, well-being, and QoL. Furthermore, home care allows patients to remain in their familiar home environment [6,7,8]. Conversely, it imposes a substantial burden on informal caregivers, who play a pivotal role in the day-to-day care of patients.

Informal caregivers of adults receiving HMV face numerous challenges, including the reorganisation of family and professional lives, restrictions on social activities, the necessity to acquire new caregiving skills, and responsibility for the well-being of loved ones. These challenges are further compounded by chronic stress and fatigue [9,10,11,12,13]. The impact of these factors on caregivers’ QoL is a significant concern.

Previous studies assessing the QoL of caregivers of ventilated patients at home have used various general measurement tools, such as the EQ-5D [14], the 36-Item Short Form Health Survey (SF-36) [15], the World Health Organisation Quality of Life Scale—Short Form (WHOQOL) [16], and the Munich Quality of Life Dimensions List [17]. Although these instruments provide valuable information on general QoL, they may not fully capture caregiving-specific experiences, such as burden, role-related strain, or positive aspects of caregiving.

The Adult Carer Quality of Life Questionnaire (AC-QoL) was developed to address this gap by assessing both negative and positive aspects of caregiving [18]. However, its applicability depends on cultural context and the specific characteristics of the caregiving population. In this context, it remains important to examine whether existing caregiver-specific instruments adequately capture QoL in clinically distinct caregiving situations, such as that of HMV.

To date, the AC-QoL has not been adapted in Poland among informal caregivers of adults receiving HMV. Moreover, previous adaptations conducted in other countries have focused mostly on caregivers of stroke survivors and individuals with cancer [19,20,21,22,23], without including this specific population. Informal caregivers of patients receiving HMV differ from other caregiver groups due to the intensity and specificity of care, including the operation of advanced medical equipment and the frequent requirement for continuous supervision. These factors may influence their quality of life and potentially affect the psychometric performance of QoL instruments [24].

The aim of this study was to translate and culturally adapt the AC-QoL for use in Poland into Polish and to perform an initial evaluation of its psychometric properties among Polish informal caregivers of adults receiving HMV.

## 2. Materials and Methods

### 2.1. Study Design and Participants

This methodological study involved two phases: (1) cross-cultural adaptation of the AC-QoL and (2) initial evaluation of its psychometric properties. Permission to adapt the instrument was obtained from Professor Stephen Joseph, the primary author of the original questionnaire [18]. The adaptation process was conducted in accordance with the guidelines proposed by Borsa, Damásio, and Bandeira [25].

The study was conducted in Poland between April 2024 and June 2025. Participants were informal caregivers of adult ventilator-assisted individuals (VAIs) living at home. A total of 203 caregivers who provided daily, unpaid care were included in the final analysis.

Inclusion criteria were: (1) being a primary, informal, unpaid caregiver of an adult VAI living at home; (2) age ≥ 18 years; (3) ability to complete questionnaires independently in Polish; (4) provision of written informed consent. Exclusion criteria were: (1) caregiving provided in institutional settings; (2) irregular or occasional caregiving; (3) professional caregiving; (4) care recipient using home oxygen therapy only.

Sealed envelopes containing participants’ information sheets, data processing information, consent forms, questionnaires, and postage-paid return envelopes were distributed through healthcare providers specialising in long-term home care for mechanically ventilated patients. In addition, recruitment was supported by the dissemination of information via social media channels and communication with patient organisations and other healthcare providers. Caregivers who initially expressed interest in the study were invited to contact the principal investigator (J.C.).

Participants were asked to read the study documents and provide informed consent before completing the questionnaires independently, with no time limitations. Participants could contact the research team by post or email to clarify any uncertainties. Upon completion, the questionnaires were returned using postage-paid envelopes, ensuring that participants did not incur any costs.

### 2.2. Ethical Considerations

The study was approved by the Ethics Committee for Scientific Research of the Jagiellonian University—Medical College, Krakow, Poland (Approval No. 118.6120.207.2023, 15 February 2024). The study was conducted in accordance with the Declaration of Helsinki and applicable data protection regulations. Participants were informed about the purpose and procedures of the study, the scope of data processing, and their right to withdraw at any stage without providing a reason. Informed consent was obtained from all study participants, including informal caregivers and experts involved in the adaptation process. All data were coded to prevent direct identification. Participants did not receive financial compensation for their involvement in the study.

### 2.3. Original Instrument

The AC-QoL is a caregiver-specific questionnaire developed to assess QoL in adults providing unpaid care. It captures both positive and negative aspects of caregiving and can be used for cross-sectional assessment as well as for evaluating changes over time.

The questionnaire consists of 40 items grouped into eight subscales: Support for Caring, Caring Choice, Caring Stress, Money Matters, Personal Growth, Sense of Value, Ability to Care, and Carer Satisfaction. Each subscale comprises five items rated on a four-point Likert scale (0—never, 1—some of the time, 2—a lot of the time, 3—always). Selected items (6–16, 19, 37, and 38) are reverse-scored. The total score ranges from 0 to 120 points, with scores of 0–40 indicating low QoL, 41–80 average QoL, and ≥81 high QoL.

The original version demonstrated very good internal consistency for the total scale (α = 0.94) and for the subscales (α = 0.78–0.89). Convergent validity was confirmed by correlations between changes in scores and self-assessment of improvement after intervention, indicating consistency between caregivers’ subjective experience and objective measurement [18,26].

The questionnaire was originally developed in English and validated in the United Kingdom [18]. It has been culturally adapted and psychometrically validated in several countries, including Italy [27], Portugal [19], Malaysia [20], South Korea [23], Iran [21], and China [22].

### 2.4. Translation and Cross-Cultural Adaptation

The AC-QoL was independently translated from English into Polish by two professional translators who were Polish native speakers and proficient in English. Both had experience in translating research instruments.

Subsequently, the authors (J.C., L.P.Ż. & M.K.) and a third independent translator, who was a Polish native speaker and had not been involved in the forward translations, compared the two versions, analysed them in terms of meaning, style, context and linguistic adequacy, and reconciled them into a single preliminary version.

The preliminary version was evaluated by a panel of eleven experts, including two psychologists, a mental health nurse, a long-term care nursing consultant, a public health specialist, a nurse experienced in home care for mechanically ventilated patients, a deputy director of a long-term care facility, a health technology assessment specialist, and three physicians specialising in anaesthesiology and intensive therapy, palliative medicine, and internal medicine, respectively. All experts were familiar with the unique nature of HMV care, while some had direct experience in the clinical management of VAIs. In the Polish healthcare system, HMV services are provided by multidisciplinary teams that must include specialists in anaesthesiology and intensive therapy or pulmonology, as well as nurses and physiotherapists. This was reflected in the composition of the expert panel, ensuring that the tool adaptation was relevant to the specific realities of Polish HMV care.

The experts assessed each item for relevance, clarity, correctness and translation equivalence, together with clarity and translation equivalence of the instruction. A four-point Likert scale was used, where ratings of 3 (quite relevant/clear/correct and equivalent) or 4 (highly relevant/clear/correct and equivalent) were considered positive responses, and ratings of 1 (not) or 2 (somewhat) were considered negative responses [28]. Experts could also suggest corrections. Although relevance and clarity are considered the most important criteria [29], correctness and comparability of the translation were also assessed, given that all experts were fluent in English.

To assess expert agreement, the item-level content validity index (I-CVI), the scale-level content validity index based on the average method (S-CVI/Ave), and the modified kappa (k*) were calculated. In accordance with the guidelines established by Polit et al. [30], the I-CVI was determined by dividing the number of experts who assigned a rating of 3 or 4 by the total number of experts. The S-CVI/Ave was calculated as the mean of the I-CVI values across all items. The probability of a chance agreement (*p_c_*) was computed using the following formula: *p_c_* = (*N*!/*A*!(*N* − *A*)!) × 0.5*^N^*, where *N* is the number of experts, and *A* is the number of experts who assigned a rating of 3 or 4. The modified kappa was calculated as: k* = (I-CVI − *p_c_*)/(1 − *p_c_*) [30]. Interpretation followed the criteria proposed by Lynn [31], with an I-CVI ≥ 0.78 and an S-CVI/Ave ≥ 0.90 considered acceptable [32]. Modified kappa values > 0.74 were interpreted as excellent [33].

The I-CVI values ranged from 0.82 to 1.00 for relevance and from 0.91 to 1.00 for clarity. The I-CVI for translation equivalence was 1.00. The S-CVI/Ave for the AC-QoL was 0.98 for relevance and 1.00 for both clarity and translation equivalence. Modified kappa values ranged from 0.81 to 1.00 for relevance, from 0.91 to 1.00 for clarity, and were 1.00 for translation equivalence. For the instruction, both the content validity index and the modified kappa were 1.00, indicating full clarity and translation equivalence. Details are presented in Supplementary Table S1.

The questionnaire was then tested in a pilot study involving 12 caregivers of adult VAIs living at home. Respondents were asked to rate the clarity and comprehensibility of each item and instruction on a four-point Likert scale. Ratings of 1 and 2 were designated as unclear or poorly understood and recoded as 0, whereas 3 and 4 were considered clear and understandable and recoded as 1 [34]. In addition, respondents were invited to provide comments via an open-ended question. The item-level face validity index (I-FVI) and the scale-level face validity index based on the average method (S-FVI/Ave) were calculated, following the recommendations for content validity described earlier [30].

The I-FVI ranged from 0.92 to 1.00, and the S-FVI/Ave was 0.99 (95% confidence interval: 0.99–1.00), indicating acceptable face validity [31,32]. For the instruction, the face validity index was 1.00. One respondent reported that the questionnaire was excessively long, while another noted that items 16 and 19 were highly similar.

In the final stage, independent conceptual back-translations were performed by an English native speaker fluent in Polish and a Polish native speaker fluent in English. The translators were not familiar with the original English version. Subsequently, the authors (J.C., L.P.Ż., & M.K.) and the translator previously involved in the reconciliation stage conducted a detailed comparison of all versions to assess the semantic and conceptual equivalence between the original version and the final Polish version. This process resulted in the final version of the questionnaire used in the study.

### 2.5. Statistical Analysis

Statistical analysis was conducted using IBM SPSS Statistics version 29.0.2.0 (Armonk, NY, USA) and JASP version 0.95.3 (JASP Team, University of Amsterdam, The Netherlands). Statistical significance was set at *p* < 0.05. Initially, the data underwent a preliminary screening to identify missing values, errors, and outliers. Cases with substantial missing data were excluded from the analysis. Prior to further analysis, items 6–16, 19, 37, and 38 were reverse-coded according to the scoring guidelines [26].

The initial required sample size was determined based on a rule of thumb, assuming a 5:1 participant-to-item ratio, with a minimum sample size of 200 participants [35], but the adequacy of the final sample size was confirmed using simulation-based recommendations. Given strong factor loadings and the number of indicators per factor, the sample size is sufficient [36].

A Confirmatory Factor Analysis (CFA) was conducted using the Weighted Least Squares Mean and Variance Adjusted (WLSMV) estimator to evaluate the fit of the original eight-factor structure of the AC-QoL in a Polish sample [37]. WLSMV was selected due to the ordinal nature of the data [38]. Prior to the analysis, the suitability of the data for CFA was evaluated, and the assumptions for ordinal indicators were considered satisfied. Model fit was assessed using commonly recommended indices, including the Comparative Fit Index (CFI), Tucker–Lewis Index (TLI), Root Mean Square Error of Approximation (RMSEA), and Standardised Root Mean Square Residual (SRMR). A model was considered to fit the data well if the values of CFI and TLI were ≥0.9, whereas values of RMSEA and SRMR ≤ 0.08 were interpreted as acceptable [39,40].

Internal consistency of the subscales and the total questionnaire was assessed using Cronbach’s alpha (α) and McDonald’s omega (ω) [41]. Values of 0.7 or higher were considered acceptable [42]. Additionally, Composite Reliability (CR) was calculated for each subscale, with values > 0.7 considered acceptable [43].

Previous studies have demonstrated associations between QoL and burden [9,14,15,16,19,27], stress [44], and satisfaction with life [27]. Therefore, it was hypothesised that the AC-QoL scores would correlate negatively with burden and stress, and positively with satisfaction with life. Convergent validity was examined using correlations between AC-QoL scores and the Caregiver Burden Scale (CBS), Perceived Stress Scale (PSS-10), and Satisfaction with Life Scale (SWLS) in their Polish versions. Convergent validity within the model was evaluated using Average Variance Extracted (AVE), with values ≥ 0.50 considered acceptable [43].

Normality was assessed using skewness, kurtosis, calculated *z*-scores, and histogram analysis. It was assumed that the absolute *z*-score for skewness and kurtosis should not exceed 3.29 to indicate normal distribution [45]. Due to the lack of normality in one subscale (Carer Satisfaction), Spearman’s rho (*ρ*) was used for correlation analysis. Correlation strength was interpreted based on the correlation coefficient, interpreted according to Schober et al. guidelines (weak ≥ 0.10, ≥0.40 moderate, ≥0.70 strong, ≥0.90 very strong) [46].

Descriptive statistics were calculated for AC-QoL items, as well as socio-demographic and clinical characteristics of the participants and care recipients. The analysis included frequencies, percentages, means, standard deviations, and minimum and maximum values. Floor and ceiling effects were calculated for the AC-QoL questionnaire, with an effect considered present if 15% or more of participants achieved the lowest (floor) or highest (ceiling) possible score at the subscale level [47].

## 3. Results

### 3.1. Characteristics of the Study Group

Approximately 2100 informal caregivers were invited to participate in the study, of whom 241 returned questionnaires (response rate ca. 11%). Thirty-eight participants were excluded. The final sample consisted of 203 informal caregivers of adult VAIs residing in 13 out of 16 administrative regions of Poland. The mean age of the informal caregivers was 59.1 ± 12.4 years. Most participants were female (80.8%) and married or living with a partner (79.3%). In most cases (80.8%), informal caregivers did not provide care for other dependents. Secondary education was the most frequently reported educational level (55.7%). The mean duration of care was 8.6 ± 10.7 years.

VAIs living at home were predominantly male (65.5%) and received either non-invasive (55.2%) or invasive (44.8%) HMV for an average duration of 5.2 ± 5.1 years. The mean score on the Activities of Daily Living (ADL) scale was 2.8 ± 2.5 points (possible range: 0–6 points). Details are presented in Table 1.

### 3.2. Factor Structure

The CFA was performed to evaluate the fit of the original eight-factor structure of the AC-QoL in the Polish sample of informal caregivers of adult VAIs living at home. The Chi-squared test (χ^2^) was significant with χ^2^(712) = 1389.99 (*p* < 0.001). Other fit indices indicated acceptable model fit (CFI = 0.954; TLI = 0.950; RMSEA = 0.069, 90% CI 0.063, 0.074; SRMR = 0.078). Standardised factor loadings ranged from 0.64 to 0.96. Each item showed the highest loading on its hypothesised factor. Details are presented in Table 2. In addition, an alternative unidimensional model was tested, which showed substantially poorer fit compared to the eight-factor solution (CFI = 0.739; TLI = 0.725; RMSEA = 0.160, 90% CI 0.156, 0.165; SRMR = 0.190).

### 3.3. Convergent Validity

Correlation analysis revealed a negative relationship between the total AC-QoL score and the CBS (*ρ* = −0.760) and PSS-10 (*ρ* = −0.682) scores. Conversely, a positive correlation was observed between the total AC-QoL score and the SWLS score (*ρ* = 0.702). A similar relationship was observed for the subscales. The strongest correlation was found between the Caring Stress subscale and the CBS score (*ρ* = −0.799), while the weakest correlation was observed between the Personal Growth subscale and the SWLS score (*ρ* = 0.261). Details are presented in Table 3.

The AVE values for all factors ranged from 0.623 to 0.843. The highest AVE was observed for the Sense of Value subscale (0.843), while the lowest was found for the Carer Satisfaction subscale (0.623).

### 3.4. Reliability

The Polish version of the AC-QoL demonstrated high internal consistency, with both Cronbach’s α and McDonald’s ω coefficients of 0.950 for the total scale. For the subscales, Cronbach’s α and McDonald’s ω ranged from 0.795 to 0.929 and 0.815 to 0.930, respectively. CR values ranged from 0.890 to 0.964 across subscales. Detailed reliability metrics are presented in Table 4.

### 3.5. Descriptive Statistics

The participants scored an average of 75.0 ± 20.1 points on the AC-QoL. The average score for each item ranged from 1.1 to 2.6. The AC-QoL total score showed no floor or ceiling effect, meaning that no participant achieved the minimum or maximum possible score. A ceiling effect was observed for one subscale (Sense of Value). Details are presented in Table 5.

## 4. Discussion

The paper presents the process of translation, cultural adaptation, and initial evidence of the psychometric properties of the Polish version of the AC-QoL (see Supplementary File S1) among informal caregivers of adult VAIs living at home in Poland [18,26]. Caring for adults receiving home mechanical ventilation requires caregivers to acquire new skills and understanding of complex care procedures. Informal caregivers must also operate specialised equipment such as ventilators, suction units, and oxygen concentrators. In addition, an informal caregiver must be constantly present to react to emergencies, which places a significant burden on informal caregivers [10]. Additionally, organisational factors, including the structure of the healthcare system in Poland, where primary responsibility for patient care often falls on the family, may influence caregiver experiences. In such a context, caregiving responsibilities may be particularly intensive, which influences informal caregivers [48].

The multidimensional structure of the AC-QoL captures both positive and negative outcomes of caring and is consistent with theoretical models of caregiver well-being, such as the stress process model [49]. For example, it reflects primary stressors (care demands and daily responsibilities), secondary strains (effects on work, social life, and mental health), and potential buffering effects of resources such as social support and coping skills [18].

The population from our study differs significantly from the group in which the original version of the tool was validated, as well as from those in other cross-cultural adaptations. The original tool was validated in a group comprising the general population of adult carers recruited from The Princess Royal Trust Carers’ Centres in the United Kingdom. Previous validations primarily focused on carers of stroke survivors [19,20,21,22], older patients with cancer [23] or children [27]. These populations differ from one another in terms of the intensity of caregiving responsibilities and the duration of caregiving, which may influence the structure of QoL dimensions.

Some characteristics of the Polish sample were similar to the original sample. The mean age of the informal caregivers in Poland (59 ± 12.4 years) was comparable to that in the UK (62 ± 11.9 years), although the mean duration of caregiving was slightly shorter (approximately 9 vs. 12 years) [18]. In our study, the mean duration of care (8.6 ± 10.7 years) exceeded the mean duration of HMV (5.2 ± 5.1 years), suggesting that informal caregivers often provide support well before ventilatory support begins, likely due to disease progression.

The cultural adaptation process included evaluation of content and face validity, both of which achieved high scores, comparable to results reported in Malaysia (S-CVI/Ave for relevance was 0.93, for clarity and comprehension was 0.97) [20] and China (S-CVI/Ave was 0.98) [22], confirming the accuracy and cultural appropriateness of the Polish version.

The original eight-factor model of the AC-QoL was tested using CFA. Although the Chi-squared test was significant (χ^2^ = 1389.99, df = 712, *p* < 0.001), which is expected in large samples, other model fit indices indicated acceptable fit (CFI = 0.954; TLI = 0.950; RMSEA = 0.069; SRMR = 0.078), with factor loadings ranging between 0.64 and 0.96. Modification indices indicated some potential cross-loadings (e.g., items 1, 29, and 31) and correlated residuals between similar items (e.g., items 34 and 35). However, modifications in questionnaire structure were not implemented due to limited theoretical justification and the risk of overfitting.

These results suggest that the original factorial structure provides an acceptable representation of the construct in this sample, although further verification in larger samples is needed, especially given some areas of misfit. All subscales of the AC-QoL were retained. This suggests that even in highly demanding clinical contexts, informal caregiver quality of life may be adequately captured using general caregiver-specific constructs. Furthermore, retaining the original structure ensures conceptual consistency with the original tool and enables cross-cultural comparison, which is essential for establishing the universal applicability of the AC-QoL across different countries.

Variations in factor structure are observed in other cultural adaptations of the AC-QoL. However, these differences may reflect not only cultural variation, but also issues related to the organisation of healthcare systems and methodological aspects of adaptation studies. The Chinese version identified the five-factor model with 31 items: Caring Benefits (13 items), Caring Stress (6 items), Caring Choice (5 items), Support for Caring (4 items), Money Matters (3 items) [22]. Similarly, the Korean adaptation confirmed a model consisting of five subscales with five items each: Money Matters, Caring Stress, Caring Choice, Ability to Care, Carer Satisfaction [23]. In the Malay adaptation, four items (16, 19, 37, and 38) were removed due to inadequate factor loadings [20]. In the Persian version of the AC-QoL, six items (11, 12, 13, 14, 29, 35) were removed, resulting in a seven-factor structure: Caring Choice, Sense of Value, Personal Growth, Support for Caring, Ability to Care, Money Matters, Carer Satisfaction [21]. It is notable that the Portuguese and Italian adaptations were the only ones to retain the original’s eight-factor structure [19,27]. These disparities suggest that the AC-QoL may be sensitive to cultural context and sample characteristics. Nevertheless, despite differences across adaptations, the AC-QoL appears to maintain stability in capturing the key dimensions of caregiver QoL.

The reliability of the Polish version of the AC-QoL, as measured by Cronbach’s α and McDonald’s ω, was 0.95 for the total scale, with the subscales ranging from 0.80 to 0.93 for Cronbach’s α and from 0.82 to 0.93 for McDonald’s ω. These values are considered good [42]. Some subscales showed very high internal consistency. High values may suggest some degree of item redundancy, indicating that some items may capture overlapping aspects of the informal caregiver experience. Inter-item polychoric correlations showed generally moderate to strong relationships within subscales, alongside weaker associations between conceptually distinct items, with higher correlations observed between conceptually similar items. This observation warrants further investigation in future studies. The original tool demonstrated high internal consistency, with Cronbach’s α of 0.94 for the total scale and 0.78–0.89 for the subscales [18]. Similarly, acceptable reliability was reported in the Chinese (α = 0.924) [22], Korean (α = 0.829) [23], Portuguese (α = 0.904) [19], Iranian (α = 0.899) [21], and Italian (α = 0.93) [27] adaptations.

The convergent validity of the Polish version of AC-QoL was assessed by significant correlations with levels of burden, stress, and satisfaction with life. A particularly strong association was observed between the Caring Stress subscale and the level of burden. In accordance with theoretical assumptions and the findings of previous studies, including prior adaptations, higher QoL among carers was associated with lower levels of burden and stress and greater life satisfaction [9,14,15,16,19,27,44]. In the Malay adaptation by Khan et al. [20], caregivers with lower stress levels achieved higher AC-QoL scores compared with their counterparts. A similar pattern was observed for caregivers with and without anxiety. The use of these instruments provides evidence of convergent validity and reflects domains closely linked to the caregiver QoL. However, generic QoL instruments were not included, as the study focused on constructs specifically associated with caregiving. Inclusion of these generic QoL instruments would have enabled broader comparisons with the general population and strengthened the interpretability of the findings. Convergent validity was confirmed in the original questionnaire by demonstrating a correlation between the AC-QoL score and self-rated improvement after intervention. The original validation study also did not examine associations with generic QoL instruments [18,26]. Convergent validity within the model was assessed using AVE. Values for all factors exceeded the recommended threshold of 0.50, indicating satisfactory convergent validity within the AC-QoL subscales model.

The analysis of the response distribution revealed a ceiling effect in one subscale (Sense of Value). This may reflect either a high perceived value associated with caregiving or a limited ability of the scale to discriminate between individuals with high scores. While ceiling effects do not necessarily indicate a lack of sensitivity, they should be interpreted with caution. This effect could be driven by psychological factors, such as finding meaning—which helps informal caregivers cope with stress, lower their burden and anxiety [50]—as well as by social factors, like socially desirable responding [51]. Nevertheless, modifying the rating scale has the potential to mitigate ceiling effects and enhance the sensitivity of the questionnaire [52]. However, this requires further research.

The AC-QoL was developed to comprehensively capture quality of life in the context of performing the multidimensional caregiver role. Accordingly, it can serve as a complementary tool to assess specific aspects of the caregiver experience. The Polish version of the AC-QoL questionnaire may be particularly useful in clinical practice and research settings. It can assist in identifying informal caregivers who require support and monitor them over time. The instrument may also help evaluate interventions aimed at improving their well-being and tailoring support for their needs.

### Limitations

This study is subject to several limitations. The most important relate to sampling and measurement design. Firstly, the convenience sampling method and voluntary participation carry the risk of self-selection bias, as individuals with greater involvement or specific experiences may have been more likely to participate. In addition, the modest sample size, although sufficient for CFA, limits the generalisability of the findings and precludes advanced analyses such as measurement invariance testing and cross-validation procedures (e.g., split-sample or bootstrapping).

Secondly, some measurement limitations should be noted. A ceiling effect observed in one subscale may limit the tool’s ability to differentiate between respondents with the highest level of the trait. Moreover, the very high internal consistency may indicate some item redundancy, which requires further investigation. Inter-item correlations also suggested generally moderate to strong associations within subscales, with some higher correlations between conceptually similar items.

Thirdly, limitations in validity and reliability assessment should be acknowledged. The study did not include widely used generic QoL instruments, which limited the scope of the convergent validity assessment. In addition, test–retest reliability was not examined, preventing evaluation of the stability results over time.

Finally, the study focused on a specific group of informal caregivers of adult VAIs living at home, which may limit the applicability of the findings to other caregiver populations. Further research in larger and independent samples is needed.

## 5. Conclusions

The Polish version of the AC-QoL demonstrates promising psychometric properties and appears to be a suitable instrument for assessing QoL among informal caregivers of adults receiving HMV. The findings indicate acceptable levels of validity and internal consistency. Despite certain limitations, the questionnaire may be useful in clinical practice and further research within this specific caregiving context. Future studies are encouraged to examine the stability of results over time, test measurement invariance, and assess applicability in more diverse caregiver populations.

## Figures and Tables

**Table 1:** Characteristics of informal caregivers and VAIs living at home (*N* = 203).

Variable		Value
Informal caregivers:		
Age (years), *mean ± SD*		59.1 ± 12.4
Gender, *n* (%)		
	Female	164 (80.8%)
	Male	39 (19.2%)
Marital status, *n* (%)		
	Married or informal partnership	161 (79.3%)
	Single (including divorced)	21 (10.3%)
	Widow/widower	21 (10.3%)
Education, *n* (%)		
	Primary education (ISCED level 1–2)	18 (8.9%)
	Secondary education (ISCED level 3–4)	113 (55.7%)
	Higher education (ISCED level 5–8)	72 (35.5%)
Employment, *n* (%)		
	Full-time	30 (14.8%)
	Part-time	18 (8.9%)
	Pensioner	101 (49.8%)
	Other, including nursing allowance	54 (26.6%)
Other persons under care, *n* (%)		
	Yes	39 (19.2%)
	No	164 (80.8%)
VAIs living at home:		
Age (years), *mean ± SD*		58.2 ± 19.8
Gender, *n* (%)		
	Female	70 (34.5%)
	Male	133 (65.5%)
Number of years in home ventilation (years), *mean ± SD*		5.2 ± 5.1
Type of home ventilation, *n* (%)		
	Invasive ventilation	91 (44.8%)
	Non-invasive ventilation	112 (55.2%)

Abbreviations: *N*—number of respondents; %—percentage of respondents; SD—standard deviation; ISCED—The International Standard Classification of Education, VAIs—ventilator-assisted individuals.

**Table 2:** Factor loadings in the Polish version of the AC-QoL (*N* = 203).

Factor/Item	Standardised Loading	SE	*z*-Value	*R* ^2^	95% Confidence Interval	
					Lower	Upper
Support for Caring						
01.	0.96	0.03	33.61	0.92	0.90	1.00
02.	0.83	0.03	29.12	0.69	0.77	0.89
03.	0.92	0.02	51.38	0.84	0.88	0.95
04.	0.96	0.01	69.30	0.92	0.93	0.99
05.	0.90	0.02	47.28	0.81	0.86	0.94
Caring Choice						
06.	0.92	0.02	50.91	0.84	0.88	0.95
07.	0.89	0.02	44.56	0.80	0.85	0.93
08.	0.87	0.03	35.14	0.76	0.82	0.92
09.	0.89	0.03	34.27	0.79	0.84	0.94
10.	0.87	0.03	33.02	0.75	0.82	0.92
Caring Stress						
11.	0.93	0.02	56.02	0.86	0.90	0.96
12.	0.92	0.02	50.16	0.85	0.88	0.96
13.	0.94	0.01	66.41	0.88	0.91	0.96
14.	0.90	0.02	49.36	0.82	0.87	0.94
15.	0.87	0.03	35.17	0.76	0.82	0.92
Money Matters						
16.	0.90	0.04	21.93	0.81	0.82	0.98
17.	0.91	0.04	22.94	0.83	0.83	0.99
18.	0.72	0.05	15.84	0.51	0.63	0.81
19.	0.79	0.04	18.88	0.63	0.71	0.88
20.	0.78	0.05	16.51	0.61	0.69	0.88
Personal Growth						
21.	0.83	0.04	21.93	0.69	0.75	0.90
22.	0.82	0.03	27.06	0.68	0.76	0.88
23.	0.93	0.02	46.94	0.88	0.90	0.98
24.	0.96	0.03	37.96	0.91	0.91	1.00
25.	0.80	0.03	23.64	0.65	0.74	0.87
Sense of Value						
26.	0.96	0.01	87.89	0.92	0.94	0.98
27.	0.96	0.01	81.77	0.93	0.94	0.99
28.	0.91	0.02	43.85	0.83	0.87	0.95
29.	0.86	0.03	25.60	0.75	0.80	0.93
30.	0.89	0.03	35.17	0.79	0.84	0.94
Ability to Care						
31.	0.89	0.04	21.37	0.78	0.80	0.97
32.	0.87	0.03	25.84	0.75	0.80	0.93
33.	0.81	0.04	23.17	0.65	0.74	0.88
34.	0.89	0.02	38.15	0.79	0.85	0.94
35.	0.78	0.04	20.53	0.61	0.70	0.85
Carer Satisfaction						
36.	0.65	0.06	10.96	0.42	0.53	0.77
37.	0.82	0.04	21.45	0.66	0.74	0.89
38.	0.64	0.06	11.27	0.41	0.53	0.75
39.	0.85	0.03	30.19	0.71	0.79	0.90
40.	0.95	0.02	50.28	0.91	0.92	0.99

Abbreviations: *N*—number of respondents; SE—standard error; *R*^2^—coefficient of determination. Note: All loadings were statistically significant at *p* < 0.001.

**Table 3:** Spearman’s rank correlation between AC-QoL subscales and the other measures (*N* = 203).

Factor	Caregiver Burden Scale	Perceived Stress Scale	Satisfaction with Life Scale
Total score	−0.760	−0.682	0.702
Support for Caring	−0.476	−0.398	0.468
Caring Stress	−0.799	−0.558	0.663
Caring Choice	−0.782	−0.525	0.584
Money Matters	−0.423	−0.446	0.490
Personal Growth	−0.279	−0.366	0.261
Sense of Value	−0.466	−0.476	0.499
Ability to Care	−0.285	−0.382	0.396
Carer Satisfaction	−0.558	−0.520	0.526

Abbreviation: *N*—number of respondents. Note: All correlations were statistically significant at *p* < 0.001 (two-tailed).

**Table 4:** Cronbach’s alpha, McDonald’s omega, and composite reliability for each AC-QoL subscale (*N* = 203).

Factor	McDonald’s ω	Cronbach’s α	Composite Reliability
Support for Caring	0.923	0.923	0.962
Caring Choice	0.920	0.920	0.949
Caring Stress	0.930	0.929	0.961
Money Matters	0.864	0.861	0.913
Personal Growth	0.904	0.901	0.941
Sense of Value	0.909	0.906	0.964
Ability to Care	0.843	0.842	0.927
Carer Satisfaction	0.815	0.795	0.890

Abbreviation: *N*—number of respondents.

**Table 5:** Descriptive statistics and floor/ceiling effects for the AC-QoL (*N* = 203).

Factor/Item		Mean ± SD	Min.–Max.	% Floor	% Ceiling
Support for Caring		9.2 ± 3.9	0–15	3.0	8.9
01.	I have a good level of emotional support	1.6 ± 0.9	0–3		
02.	My needs as a carer are considered by professionals	1.7 ± 0.9	0–3		
03.	I am happy with the professional support that is provided to me	2.0 ± 0.9	0–3		
04.	I feel able to get the help and information I need	2.0 ± 0.9	0–3		
05.	I have all the practical support I need	1.9 ± 0.9	0–3		
Caring Choice		8.5 ± 4.2	0–15	5.4	6.9
06.	I feel that my life is on hold because of caring	1.7 ± 0.9	0–3		
07.	My social life has suffered because of caring	1.7 ± 0.9	0–3		
08.	I feel I have less choice about my future due to caring	1.5 ± 1.0	0–3		
09.	I feel I have no control over my own life	2.0 ± 1.0	0–3		
10.	Caring stops me doing what I want to do	1.6 ± 0.9	0–3		
Caring Stress		8.9 ± 4.0	0–15	4.4	7.9
11.	I feel depressed due to caring	1.9 ± 0.9	0–3		
12.	I feel worn out as a result of caring	1.6 ± 0.9	0–3		
13.	I am mentally exhausted by caring	1.9 ± 0.8	0–3		
14.	I am physically exhausted by caring	1.6 ± 0.9	0–3		
15.	I feel stressed as a result of caring	1.9 ± 0.8	0–3		
Money Matters		7.6 ± 3.7	0–15	2.5	4.9
16.	I worry about going into debt	2.2 ± 0.8	0–3		
17.	I feel satisfied with my financial situation	1.2 ± 0.9	0–3		
18.	I am able to save for a rainy day	1.1 ± 0.9	0–3		
19.	I worry about money	1.7 ± 0.9	0–3		
20.	There is enough money in our house to pay for the things we need	1.4 ± 1.0	0–3		
Personal Growth		8.6 ± 3.6	0–15	1.0	6.4
21.	I have become a more tolerant person through my caring role	1.9 ± 0.8	0–3		
22.	Because of caring, I have learnt a lot about myself	1.9 ± 0.9	0–3		
23.	Because of caring, I feel that I have grown as a person	1.6 ± 0.9	0–3		
24.	I have experienced many positive things through caring	1.5 ± 0.8	0–3		
25.	I feel that I have become a better person by caring	1.8 ± 0.9	0–3		
Sense of Value		10.6 ± 3.7	0–15	0.5	19.2
26.	I feel valued by the person I am looking after	2.1 ± 0.9	0–3		
27.	The person I look after respects me for what I do	2.2 ± 0.9	0–3		
28.	The person I look after makes me feel good about myself	2.0 ± 0.9	0–3		
29.	I get a lot from the person I am looking after	1.7 ± 0.9	0–3		
30.	I have a good relationship with the person I am caring for	2.5 ± 0.7	0–3		
Ability to Care		11.0 ± 2.6	4–15	0.0	12.8
31.	I am satisfied with my performance as a carer	2.0 ± 0.7	0–3		
32.	I can take care of the needs of the person I am caring for	2.4 ± 0.6	1–3		
33.	I feel I am able to make the life of the person I am looking after better	2.2 ± 0.7	0–3		
34.	I can manage most situations with the person I care for	2.2 ± 0.6	1–3		
35.	I am able to deal with a difficult situation	2.2 ± 0.6	1–3		
Carer Satisfaction		10.7 ± 3.0	1–15	0.0	11.3
36.	Caring is important to me	2.6 ± 0.6	1–3		
37.	I resent having to be a carer	2.4 ± 0.8	0–3		
38.	I feel frustrated with the person I am caring for	2.5 ± 0.7	0–3		
39.	I enjoy being a carer	1.7 ± 1.0	0–3		
40.	I am satisfied with my life as a carer	1.6 ± 0.9	0–3		
Total scale		75.0 ± 20.1	18–118	0.0	0.0

Abbreviation: *N*—number of respondents; SD—standard deviation; Min.–Max.—minimum–maximum; % Floor—percentage of respondents with the lowest possible score; % Ceiling—percentage of respondents with the highest possible score.

## Data Availability

The original contributions presented in this study are included in the Supplementary Materials. Further inquiries can be directed to the corresponding author.

## Supplementary Materials

The following supporting information can be downloaded at: https://www.mdpi.com/article/10.3390/jcm15103587/s1. Table S1: Relevance and clarity of the AC-QoL; File S1: The Polish version of the Adult Carer Quality of Life Questionnaire; File S2: Dataset; File S3: Inter-item polychoric correlation matrix of the AC-QoL items.

## Abbreviations

The following abbreviations are used in this manuscript:

% Ceiling	Percentage of respondents with the highest possible score
% Floor	Percentage of respondents with the lowest possible score
AC-QoL	Adult Carer Quality of Life Questionnaire
α	Cronbach’s alpha
AVE	Average Variance Extracted
ca.	Approximately
CBS	Caregiver Burden Scale
CFA	Confirmatory Factor Analysis
CFI	Comparative Fit Index
CR	Composite Reliability
df	Degrees of freedom
e.g.,	For example
HMV	Home Mechanical Ventilation
I-CVI	Item-level Content Validity Index
I-FVI	Item-level Face Validity Index
ISCED	International Standard Classification of Education
*k**	Modified kappa
Min.–Max.	Minimum–maximum
*N*	Number of respondents
PSS-10	Perceived Stress Scale
QoL	Quality of Life
*R* ^2^	Coefficient of determination
RMSEA	Root Mean Square Error of Approximation
S-CVI/Ave	Scale-level Content Validity Index based on the average method
S-FVI/Ave	Scale-level Face Validity Index based on the average method
SD	Standard Deviation
SE	Standard Error
SRMR	Standardised Root Mean Square Residual
SWLS	Satisfaction with Life Scale
TLI	Tucker–Lewis Index
VAIs	Ventilator-Assisted Individuals
WLSMV	Weighted Least Squares Mean and Variance Adjusted
χ^2^	Chi-squared test
*ρ*	Spearman’s rho
ω	McDonald’s omega
