# Supplementary material for: Cross-Cultural Adaptation and Initial Psychometric Evaluation of the Adult Carer Quality of Life Questionnaire (AC-QoL) Among Informal Caregivers of Adults Receiving Home Mechanical Ventilation in Poland
Source: J Clin Med. 2026 May 7;15(10):3587. doi: 10.3390/jcm15103587 (PMC13207568; doi:10.3390/jcm15103587)
Supplement: Supplementary file 1 [file jcm-15-03587-s001.zip › Supplementary File S1 - Polish AC-QoL.pdf]

KWESTIONARIUSZ JAKOŚCI ŻYCIA OPIEKUNA (AC-QoL)

Jak wypełnić kwestionariusz?

W tym kwestionariuszu zawarto pytania dotyczące różnych aspektów Twojego życia jako opiekuna. Proszę pomyśleć o swoich doświadczeniach z ostatnich dwóch tygodni i zaznaczyć odpowiednie pole obok każdego stwierdzenia. Nie ma dobrych ani złych odpowiedzi. Interesuje nas tylko to, jak wygląda Twoje życie jako opiekun. Ankieta nie powinna zająć więcej niż 10 minut. Proszę odpowiedzieć na wszystkie pytania tak szczerze, jak to tylko możliwe.

Wsparcie w opiece		Nigdy	Czasami	Często	Zawsze
01.	Mam dobry poziom wsparcia emocjonalnego	☐	☐	☐	☐
02.	Moje potrzeby jako opiekunki/a są brane pod uwagę przez profesjonalistów	☐	☐	☐	☐
03.	Jestem zadowolona/y ze wsparcia otrzymywanego ze strony profesjonalistów	☐	☐	☐	☐
04.	Czuję, że mogę otrzymać pomoc i informacje, których potrzebuję	☐	☐	☐	☐
05.	Mam wszelkie wsparcie praktyczne, jakiego potrzebuję	☐	☐	☐	☐

Opieka jako wybór		Nigdy	Czasami	Często	Zawsze
06.	Czuję, że moje życie jest w zawieszeniu w związku ze sprawowaniem opieki	☐	☐	☐	☐
07.	Moje życie towarzyskie cierpi z powodu sprawowania opieki	☐	☐	☐	☐
08.	Czuję, że moje wybory dotyczące przyszłości zostały ograniczone ze względu na sprawowanie opieki	☐	☐	☐	☐
09.	Czuję, że nie mam kontroli nad swoim życiem	☐	☐	☐	☐
10.	Sprawowanie opieki uniemożliwia mi robienie tego, co chcę	☐	☐	☐	☐

Stres związany ze sprawowaniem opieki		Nigdy	Czasami	Często	Zawsze
11.	Czuję się przygnębiona/y w związku ze sprawowaniem opieki	☐	☐	☐	☐
12.	Czuję się wyczerpana/y sprawowaniem opieki	☐	☐	☐	☐
13.	Jestem psychicznie wykończona/y sprawowaniem opieki	☐	☐	☐	☐
14.	Jestem fizycznie wykończona/y sprawowaniem opieki	☐	☐	☐	☐
15.	Czuję się zestresowana/y w wyniku sprawowania opieki	☐	☐	☐	☐

Kwestie finansowe		Nigdy	Czasami	Często	Zawsze
16.	Martwię się, że wpadnę w długi	☐	☐	☐	☐
17.	Jestem zadowolona/y z mojej sytuacji finansowej	☐	☐	☐	☐
18.	Jestem w stanie odłożyć pieniądze na czarną godzinę	☐	☐	☐	☐
19.	Martwię się o pieniądze	☐	☐	☐	☐
20.	Mamy wystarczająco dużo pieniędzy w naszym gospodarstwie domowym, aby płacić za wszystkie potrzebne nam rzeczy	☐	☐	☐	☐

Rozwój osobisty		Nigdy	Czasami	Często	Zawsze
21.	Odkąd pełnię rolę opiekunki/a stałam/em się bardziej tolerancyjna/y	☐	☐	☐	☐
22.	Dzięki sprawowaniu opieki nauczyłam/em się wiele o sobie	☐	☐	☐	☐
23.	Czuję, że dzięki sprawowaniu opieki rozwinęłam/em się jako osoba	☐	☐	☐	☐
24.	Doświadczyłam/em wielu pozytywnych rzeczy w związku ze sprawowaniem opieki	☐	☐	☐	☐
25.	Czuję, że dzięki sprawowaniu opieki, stałam/em się lepszym człowiekiem	☐	☐	☐	☐

Poczucie wartości		Nigdy	Czasami	Często	Zawsze
26.	Czuję się doceniana/y przez osobę, którą się opiekuję	☐	☐	☐	☐
27.	Osoba, którą się opiekuję, szanuje mnie za to, co robię	☐	☐	☐	☐
28.	Osoba, którą się opiekuję, sprawia, że dobrze o sobie myślę	☐	☐	☐	☐
29.	Opieka wiele mi daje	☐	☐	☐	☐
30.	Mam dobrą relację z osobą, którą się opiekuję	☐	☐	☐	☐

Zdolność do sprawowania opieki		Nigdy	Czasami	Często	Zawsze
31.	Jestem zadowolona/y z tego, jak sprawuję opiekę	☐	☐	☐	☐
32.	Potrafię zadbać o potrzeby osoby, którą się opiekuję	☐	☐	☐	☐
33.	Czuję, że jestem w stanie sprawić, że życie osoby, którą się opiekuję, staje się lepsze	☐	☐	☐	☐
34.	Potrafię poradzić sobie z większością sytuacji dotyczących osoby, którą się opiekuję	☐	☐	☐	☐
35.	Potrafię poradzić sobie w trudnej sytuacji	☐	☐	☐	☐

Satysfakcja opiekunki/opiekuna		Nigdy	Czasami	Często	Zawsze
36.	Sprawowanie opieki jest dla mnie ważne	☐	☐	☐	☐
37.	Jestem rozgoryczona/y tym, że muszę być opiekunką/em	☐	☐	☐	☐
38.	Czuję się sfrustrowana/y osobą, którą się opiekuję	☐	☐	☐	☐
39.	Lubię być opiekunką/em	☐	☐	☐	☐
40.	Jestem zadowolona/y ze swojego życia jako opiekuna	☐	☐	☐	☐

OBLICZENIE WYNIKU

Obliczenie wyniku:

Nigdy	0 pkt.
Czasami	1 pkt.
Często	2 pkt.
Zawsze	3 pkt.

Pozycje 6, 7, 8, 9, 10, 11, 12, 13, 14, 15, 16, 19, 37, 38 są punktowane odwrotnie:

Nigdy	3 pkt.
Czasami	2 pkt.
Często	1 pkt.
Zawsze	0 pkt.

Aby uzyskać wynik całkowity, należy zsumować punkty ze wszystkich pozycji kwestionariusza. Im wyższy wynik, tym wyższy poziom jakości życia.

Podskale:
Na wynik każdej podskali składa się suma punktów uzyskanych w przypisanych do niej pozycjach kwestionariusza:

Wsparcie w opiece	1, 2, 3, 4, 5
Opieka jako wybór	6, 7, 8, 9, 10
Stres związany ze sprawowaniem opieki	11, 12, 13, 14, 15
Kwestie finansowe	16, 17, 18, 19, 20
Rozwój osobisty	21, 22, 23, 24, 25
Poczucie wartości	26, 27, 28, 29, 30
Zdolność do sprawowania opieki	31, 32, 33, 34, 35
Satysfakcja opiekunki/opiekuna	36, 37, 38, 39, 40
