# Supplementary material for: Cross-Cultural Adaptation and Initial Psychometric Evaluation of the Adult Carer Quality of Life Questionnaire (AC-QoL) Among Informal Caregivers of Adults Receiving Home Mechanical Ventilation in Poland
Source: J Clin Med. 2026 May 7;15(10):3587. doi: 10.3390/jcm15103587 (PMC13207568; doi:10.3390/jcm15103587)
Supplement: Supplementary file 1 [file jcm-15-03587-s001.zip › Supplementary Table S1.pdf]

Supplementary Table S1. Relevance and clarity of the AC-QoL (N=11).

Item	Relevance					Clarity				
	**Agmt.**	**I-CVI**	p_c	$k*$	**Interpr.**	**Agmt.**	**I-CVI**	p_c	$k*$	**Interpr.**
Subscale: Support for Caring										
01	10/11	0.91	0.0054	0.91	Exc	11/11	1.00	0.0005	1.00	Exc
02	11/11	1.00	0.0005	1.00	Exc	11/11	1.00	0.0005	1.00	Exc
03	11/11	1.00	0.0005	1.00	Exc	11/11	1.00	0.0005	1.00	Exc
04	11/11	1.00	0.0005	1.00	Exc	11/11	1.00	0.0005	1.00	Exc
05	11/11	1.00	0.0005	1.00	Exc	10/11	0.91	0.0054	0.91	Exc
Subscale: Caring Choice										
06	11/11	1.00	0.0005	1.00	Exc	11/11	1.00	0.0005	1.00	Exc
07	11/11	1.00	0.0005	1.00	Exc	11/11	1.00	0.0005	1.00	Exc
08	10/11	0.91	0.0054	0.91	Exc	11/11	1.00	0.0005	1.00	Exc
09	11/11	1.00	0.0005	1.00	Exc	11/11	1.00	0.0005	1.00	Exc
10	10/11	0.91	0.0054	0.91	Exc	11/11	1.00	0.0005	1.00	Exc
Subscale: Caring Stress										
11	10/11	0.91	0.0054	0.91	Exc	11/11	1.00	0.0005	1.00	Exc
12	11/11	1.00	0.0005	1.00	Exc	11/11	1.00	0.0005	1.00	Exc
13	11/11	1.00	0.0005	1.00	Exc	11/11	1.00	0.0005	1.00	Exc
14	10/11	0.91	0.0054	0.91	Exc	11/11	1.00	0.0005	1.00	Exc
15	11/11	1.00	0.0005	1.00	Exc	11/11	1.00	0.0005	1.00	Exc
Subscale: Money Matters										
16	9/11	0.82	0.0269	0.81	Exc	11/11	1.00	0.0005	1.00	Exc
17	11/11	1.00	0.0005	1.00	Exc	11/11	1.00	0.0005	1.00	Exc
18	11/11	1.00	0.0005	1.00	Exc	11/11	1.00	0.0005	1.00	Exc
19	10/11	0.91	0.0054	0.91	Exc	11/11	1.00	0.0005	1.00	Exc
20	11/11	1.00	0.0005	1.00	Exc	11/11	1.00	0.0005	1.00	Exc
Subscale: Personal Growth										
21	11/11	1.00	0.0005	1.00	Exc	11/11	1.00	0.0005	1.00	Exc
22	11/11	1.00	0.0005	1.00	Exc	11/11	1.00	0.0005	1.00	Exc
23	11/11	1.00	0.0005	1.00	Exc	11/11	1.00	0.0005	1.00	Exc
24	11/11	1.00	0.0005	1.00	Exc	11/11	1.00	0.0005	1.00	Exc
25	11/11	1.00	0.0005	1.00	Exc	11/11	1.00	0.0005	1.00	Exc
Subscale: Sense of Value										
26	11/11	1.00	0.0005	1.00	Exc	11/11	1.00	0.0005	1.00	Exc
27	11/11	1.00	0.0005	1.00	Exc	11/11	1.00	0.0005	1.00	Exc
28	11/11	1.00	0.0005	1.00	Exc	11/11	1.00	0.0005	1.00	Exc
29	11/11	1.00	0.0005	1.00	Exc	11/11	1.00	0.0005	1.00	Exc
30	11/11	1.00	0.0005	1.00	Exc	11/11	1.00	0.0005	1.00	Exc
Subscale: Ability to Care										
31	11/11	1.00	0.0005	1.00	Exc	11/11	1.00	0.0005	1.00	Exc
32	11/11	1.00	0.0005	1.00	Exc	11/11	1.00	0.0005	1.00	Exc
33	11/11	1.00	0.0005	1.00	Exc	11/11	1.00	0.0005	1.00	Exc
34	11/11	1.00	0.0005	1.00	Exc	11/11	1.00	0.0005	1.00	Exc
35	11/11	1.00	0.0005	1.00	Exc	11/11	1.00	0.0005	1.00	Exc
Subscale: Carer Satisfaction										
36	11/11	1.00	0.0005	1.00	Exc	11/11	1.00	0.0005	1.00	Exc
37	10/11	0.91	0.0054	0.91	Exc	11/11	1.00	0.0005	1.00	Exc
38	10/11	0.91	0.0054	0.91	Exc	11/11	1.00	0.0005	1.00	Exc
39	11/11	1.00	0.0005	1.00	Exc	11/11	1.00	0.0005	1.00	Exc
40	11/11	1.00	0.0005	1.00	Exc	11/11	1.00	0.0005	1.00	Exc
**S-CVI/Ave**	**0.98** (95% CI: 0.96-0.99)					**1.00** (95% CI: 0.99-1.00)				

Abbreviations: N – number of experts; Agmt. – agreement; Interpr. – interpretation; Exc – excellent; I-CVI – item-level content validity index; S-CVI/Ave – scale-level content validity index; p_c – probability of a chance occurrence; $k*$ – modified kappa; 95% CI – 95% confidence interval.
